# Glucose oxidase kinetics using MnO_2_ nanosheets: confirming Michaelis–Menten kinetics and quantifying decreasing enzyme performance with increasing buffer concentration†

**DOI:** 10.1039/d1na00311a

**Published:** 2021-05-17

**Authors:** Mahip Singh, Ungku Zoe Anysa Ungku Faiz, Steven Gravelsins, Yoshinori Suganuma, Nicholas Konstantine Kotoulas, Mark Croxall, Ahlia Khan-Trottier, Cynthia Goh, Al-Amin Dhirani

**Affiliations:** Department of Chemistry, University of Toronto Ontario Canada M5S 3H6 a.dhirani@utoronto.ca; Department of Biochemistry, University of Toronto Ontario Canada M5S 3H6; Department of Physics, University of Toronto Toronto Ontario Canada M5S 3H6

## Abstract

MnO_2_ nanosheets and ultraviolet-visible (UV-Vis) absorbance spectroscopy are used to study glucose oxidase (GOx) kinetics. Glucose oxidation by GOx produces H_2_O_2_, which rapidly decomposes the nanosheets and reduces their absorption. This direct approach for monitoring glucose oxidation enables simpler, real time kinetics analysis compared to methods that employ additional enzymes. Using this approach, the present study confirms that GOx kinetics is consistent with the Michaelis–Menten (MM) model, and reveals that the MM constant increases by an order of magnitude with increasing buffer concentration. Since larger MM constants imply higher enzyme substrate concentrations are required to achieve the same rate of product formation, increasing MM constants imply decreasing enzyme performance. These results demonstrate the facility of using MnO_2_ nanosheets to study GOx kinetics and, given the widespread applications of enzymes with buffers, the important sensitivity of enzyme–buffer systems on buffer concentration.

## Abstract 2

Glucose oxidase, GOx, kinetics probed using ultraviolet-visible absorbance of MnO_2_ nanosheets confirm Michaelis–Menten (MM) kinetics and reveal a 10-fold increase in MM constant with increasing buffer concentration.

## Graphical abstract

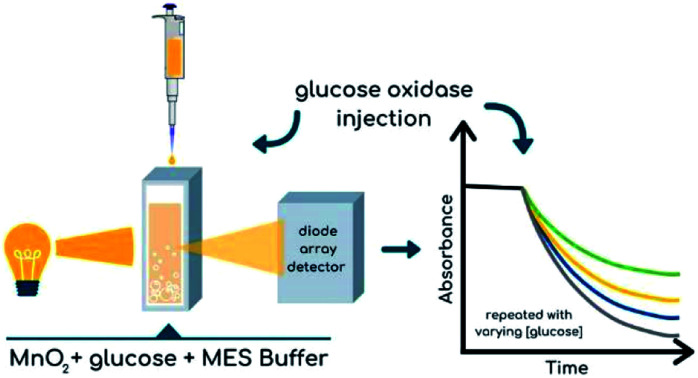

## 1. Introduction

**Scheme 1:**
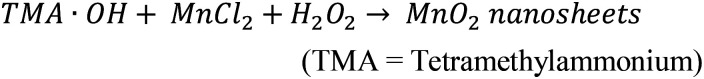


**Scheme 2:**
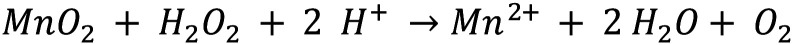


**Scheme 3:**
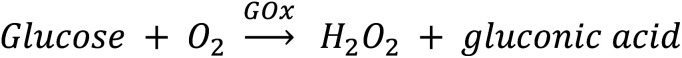


Manganese dioxide (MnO_2_) can be used for a variety of applications such as biosensing in biomedical applications,^1^ energy storage in batteries,^2^ water decontamination at low temperatures,^3^ catalysis with high activity and thermal stability,^4^ and hydrogen peroxide decomposition to oxygen and water.^5^ Recently, studies have reported that aqueous solutions of MnO_2_ nanosheets can be easily synthesized according to Scheme 1.^6^ Nanosheets are characterized by nanometre-scale (potentially atomic-scale) thicknesses and micrometre-scale lateral dimensions.^6^ Other materials that can be isolated as nanosheets include graphite, molybdenum disulphide and boron nitride.^7^ Increasing recent interest in such nanosheet forms of materials has arisen due to significant differences in chemical and physical properties exhibited by nanosheets compared to their bulk counterparts; for example, nanosheets possess much higher specific surface areas and can even exhibit distinct band structures and spectral behaviour. MnO_2_ nanosheets, for example, exhibit a prominent ultraviolet-visible (UV-Vis) spectral peak around 386 nm.^6^ Recent studies have reported that the peak decreases in the presence of H_2_O_2_ as H_2_O_2_ acts as an oxidizing agent^6^ and rapidly decomposes the nanosheets as per Scheme 2.^8,9^ This spectral behaviour suggests a potential application for monitoring glucose oxidation since H_2_O_2_ is a product of this reaction, as seen in Scheme 3.^9^ Potential biological and medical applications include detecting H_2_O_2_ in cells and monitoring health/detecting diseases^10^ in low resource environments.^9,11^

MnO_2_ nanosheets also potentially provide a new tool for studying glucose oxidase (GOx) kinetics in real time. As per Scheme 3, GOx catalyzes oxidation of glucose and the production of H_2_O_2_, which can be monitored *via* MnO_2_ decomposition in real time using UV-Vis spectroscopy. The present study confirms that this is possible and finds that the reaction between MnO_2_ and H_2_O_2_ occurs more rapidly than that of glucose oxidation. A resulting advantage of using this approach is that analysis of GOx kinetics is greatly simplified compared to approaches that employ additional enzymes, such as horseradish peroxidase (HRP), to detect H_2_O_2_ by a colour change.^12^ Previous studies have reported other methods for quantifying H_2_O_2_, for example, using composite materials and electrochemistry or conductivity measurements.^13,14^ However, application of these methods to study GOx kinetics has not been demonstrated.

Understanding enzyme kinetics is important as enzymes are common biocatalysts and are widely used in industrial processes.^15–19^ GOx is an ideal candidate for studying enzyme kinetics given its stability and the ease of the method presented here.^12^ Further, GOx itself has been used in a range of applications such as detecting and managing diabetes *via* glucose monitors functionalized with HRP,^11,12^ modifying dairy texture,^20^ and removing glucose from eggs to extend shelf-life.^21^ We apply the present approach to monitor GOx kinetics in real time and test the applicability of the Michaelis–Menten (MM) kinetics model to GOx. We find that plots of initial rate *vs.* initial glucose concentration fit well to predictions of this model. We also confirm a hypothesis that GOx kinetics is affected by buffer concentration by measuring MM constants over a wide range of buffer concentrations. Enzyme activity is known to be affected by pH, and hence buffers are widely used to maintain constant pH. At the same time, the total ion concentration in solution will affect charge screening, protein structure and enzyme–substrate interactions; thus, the presence and concentration of the buffer itself, which alters the ion concentration of the solution, will have an effect on enzyme activity. For example, polar media are known to disrupt protein structure and influence protein function, as shown previously, and are expected to affect enzyme activity.^22^ This has significant implications given ubiquitous applications of enzyme–buffer systems; however, the systematic quantification of any potential effects of buffer concentration on the function of enzymes has not been documented in the literature.

## 2. Experimental

### Chemicals

Chemicals are purchased from Sigma Aldrich, Millipore, Fluka and ACP Chemicals. All chemicals were used as received. Deionized- (DI-) water is obtained in-house (18.2 MOhm cm^−1^ at 25 °C).

### Synthesis, purification and isolation of MnO_2_ nanosheets

MnO_2_ nanosheets are synthesized using published methods.^6,9^ 2.0 mL of H_2_O_2_ (30% by weight in water, ACS Grade, Sigma Aldrich, 216763 500 mL bottle) is added to 3.94 mL of aqueous tetramethylammonium hydroxide (TMA·OH, 25% by weight in water, Sigma Aldrich, 331635 250 mL bottle) in 14.1 mL of H_2_O. This mixture is added within 15 seconds to 0.2845 g of aqueous manganese chloride, MnCl_2_ (≥99%, trace metal basis, Sigma Aldrich 244589 10 g bottle) in 10.0 mL of water. The mixture exhibits effervescence for roughly 5 minutes and becomes a dark brown suspension. The mixture is stirred overnight at room temperature in air and can be stored in a refrigerator before further processing.

To remove larger particles, the suspension is sonicated (Branson 1510 sonicator) for 2 hours and centrifuged at ∼3000 rpm for 40 minutes. The supernatant is then vacuum filtered and, to facilitate washing, its volume is reduced to ∼5 mL by heating to 73 °C and vacuum pumping using a rotary evaporator. The nanosheets in solution are precipitated by adding 10 mL ethanol (95% in water) and centrifuging for 20 minutes at ∼3000 rpm. The precipitate is washed with ethanol and redispersed in DI-water. The precipitation and washing steps are repeated using 20 mL ethanol and centrifuging for 30 minutes. The supernatant is removed, leaving behind a small amount of brown viscous precipitate. The precipitate, which contains the MnO_2_ nanosheets, is washed again with methanol and stored at 5 °C under argon in a parafilm-sealed vial. To test reproducibility, the synthesis is performed twice, and products from both syntheses are used in kinetic measurements reported below.

### Kinetic measurements

MnO_2_ nanosheet solutions are freshly prepared from precipitate on the same day that kinetic measurements are performed. 0.0075 g of MnO_2_ precipitate is dispersed in 20 mL DI-water, sonicated for ∼20 minutes and centrifuged for ∼25 minutes. The supernatant containing nanosheets is transferred to a clean vial, and the precipitate is discarded. The nanosheet solution is diluted as needed to ensure the absorbance at the beginning of each kinetic measurement is approximately 0.9. Various stock solutions are prepared and diluted as needed: 19.52 g of 2-(*N*-morpholino)ethane sulfonic acid (MES buffer, ULTROL grade, Millipore 475893, 100 g bottle) in 80 mL of DI-water, 0.0075 g of GOx (from *Aspergillus niger*, ∼135 U mg^−1^, Sigma Aldrich G7141) in 1.0 mL of DI-water, and 40 mM of β-d-glucose (>99%, HPLC grade, Fluka Biochemika) in DI-water. The DI-water is exposed to ambient atmosphere to allow oxygen diffusion. MES buffer and glucose (with concentrations as described below) as well as MnO_2_ nanosheets (diluted as needed to ensure an initial absorbance of ∼0.8–1.0 at 384 nm) are combined in a quartz cuvette for UV-Vis spectroscopy (Lambda 25 UV/Vis Spectrometer). The spectrometer's lid is opened after 30 seconds to inject GOx, the solution is stirred at the start of each kinetic run, and then the lid is closed. A cardboard covered in black electrical tape is used as a homemade cover to reduce ambient light reaching the spectrometer's detector while the spectrometer's lid is open. The homemade cover has a hole cut to allow access to the cuvette and is left in place throughout the kinetic run.

### Nanosheet characterization

MnO_2_ nanosheets are imaged using scanning electron microscopy (SEM, Hitachi S-5200, operated at 5.0 kV) and atomic force microscopy (AFM, JPK Nanowizard II, tapping mode). Energy dispersive spectra (EDS) were obtained using an ESEM (FEI Quanta FEG 250) operated in high vacuum mode at 10 keV. Samples for SEM are prepared by sonicating nanosheets in water, diluting and drop-casting the solution onto Carbon Type-A TEM grids and drying the grids in air. Samples for AFM are prepared by drop-casting nanosheet solution on freshly cleaved mica and drying under ambient conditions (30% humidity) overnight for imaging the following day. These samples are imaged using a silicon cantilever (NCHR-20, NanoWorld) with a force constant of 40 N m^−1^. The integral and proportional gains are set to 10 and 0.002, respectively.

## 3. Results and discussion

**Fig. 1:**
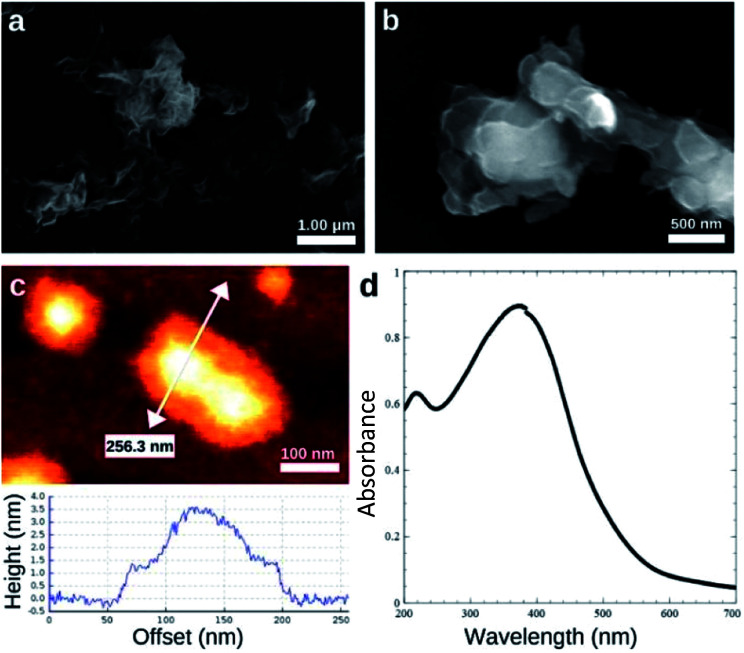
Characterization of MnO_2_ nanosheets. (a) Low and (b) high resolution SEM images. (c) AFM image obtained in tapping mode and height profile across the line shown. (d) UV/Vis spectrum of MnO_2_ nanosheets in water.

**Fig. 2:**
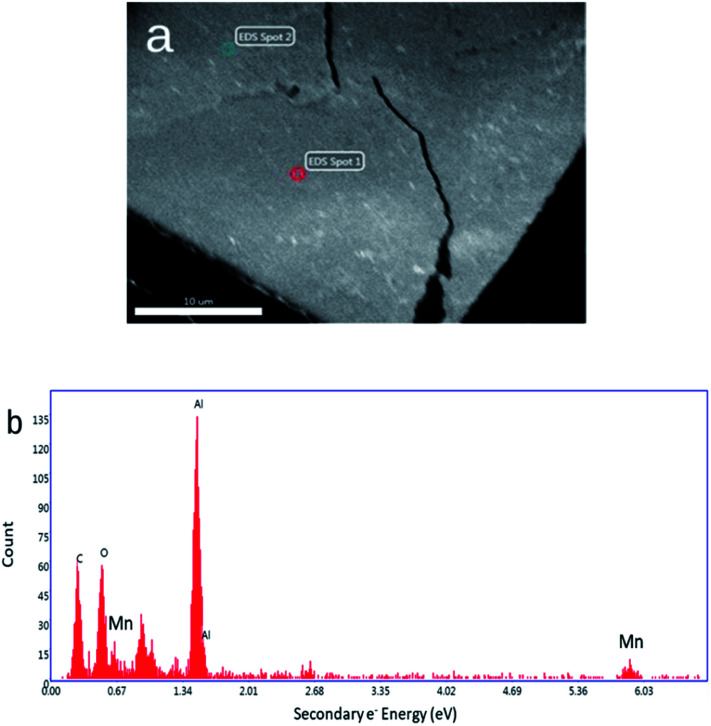
(a) ESEM image of a thick MnO_2_ film. (b) Energy dispersive spectrum obtained at Spot 1 shown in Fig. 2a (a spectrum obtained at Spot 2 is shown in Fig. S3†).

SEM and AFM images of nanosheets are shown in Fig. 1a–c. SEM images in Fig. 1a and b show that the nanosheets can be multi-layered, and that their lateral size is micron in scale. The nanosheets can appear as aggregates (Fig. 1a) potentially generated by drop-casting and drying steps. AFM images (Fig. 1c) exhibit steps and confirm that the nanosheets can be multilayered.^9^ The height profile indicates that the thickness of each layer is ∼1.2–1.5 nm, and the total thickness of the multilayer shown is ∼3.5 nm. For comparison, a previous study^6^ reported a value of 1.02 nm for the thickness of a MnO_2_ monolayer. Fig. 1d shows a UV-Vis absorbance spectrum of nanosheets in DI-water. UV-Vis spectra consistently exhibit absorption over a broad range of wavelengths and a peak around 375–382 nm, which can be ascribed to d–d transitions^6^ in Mn ions as previously reported. Previous studies^8,9^ have also reported that nanosheet absorbance in this wavelength range decreases in the presence of H_2_O_2_ in direct proportion to the amount of H_2_O_2_. ESEM images are shown in Fig. 2a and S2.† Complementary EDS spectra taken at two locations shown in Fig. 2a. Characteristic Mn peaks appear at 5.9 eV (Kα) and 0.637 eV (Lα) and confirm the presence of Mn in the sample (Fig. 2b and S3†). Al peaks can be attributed to a sample holder and C peaks to a TEM grid.

**Fig. 3:**
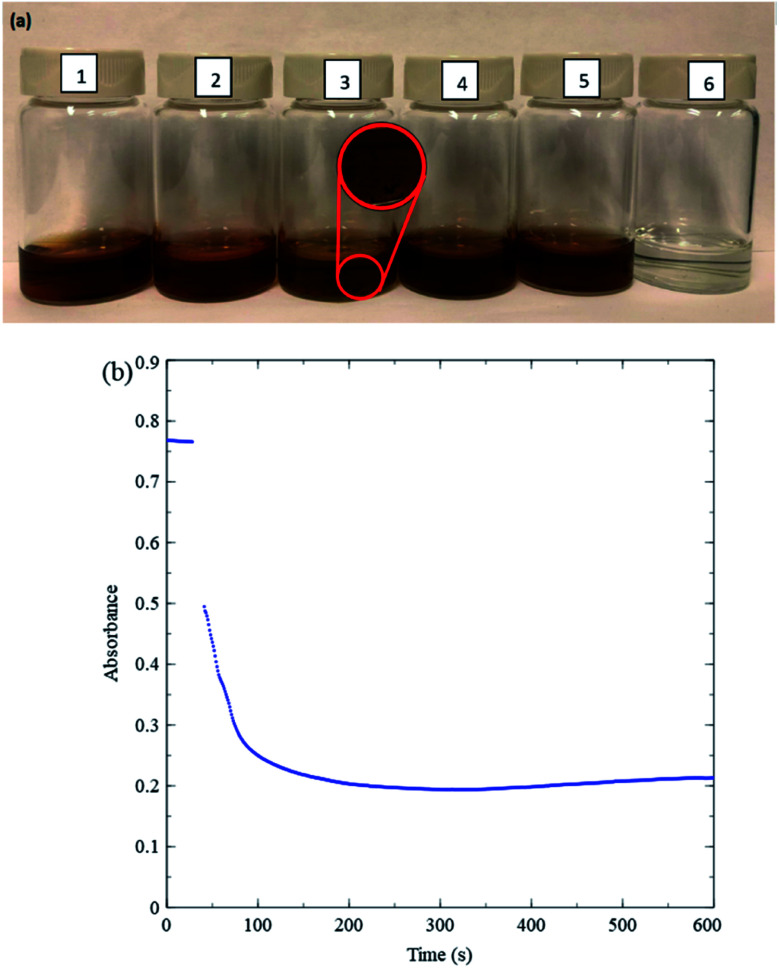
(a) Photographs of various nanosheet solutions in DI-water 15 min. after preparation (1) MnO_2_; (2) MnO_2_ and HCl; (3) MnO_2_ and NaCl; (4) MnO_2_, MES buffer and glucose; (5) MnO_2_, MES buffer, and gluconic acid; (6) MnO_2_, MES buffer, gluconic acid and H_2_O_2_. (b) Absorbance of the nanosheets in DI-water at 384 nm *vs.* time upon injecting H_2_O_2._ Near the ∼30 s mark, about 15 s of data have been omitted due to absorbance fluctuations caused by opening the spectrometer lid, injecting H_2_O_2_ and stirring. Initial reaction rate approx. 0.022 s^−1^.

Fig. 3a pictures a general overview of changes in nanosheet colour in a variety of solutions with or without H_2_O_2_. Fig. 3a(1) shows a nanosheet solution in deionized water (relatively concentrated here for improved visibility), and Fig. 3a(6) shows a solution with the same nanosheet concentration 15 min after adding H_2_O_2_. The H_2_O_2_ causes a clear loss of colour. Fig. 3b shows a time evolution of a drop in absorbance of diluted nanosheet solution at 384 nm upon adding H_2_O_2_. The absorbance of the nanosheets drops significantly and rapidly upon addition of the H_2_O_2_. This serves as an important control test because glucose oxidation proceeds more slowly (the rates are discussed below). The nanosheet concentration is typical, and the 10 mM H_2_O_2_ concentration used for this test is an effective upper bound for the maximum possible H_2_O_2_ concentration available in any kinetic measurement performed in this study. Therefore, the rate-limiting step is the glucose oxidation reaction, and measurements of absorbance of nanosheets can be used as a straightforward means for analyzing kinetics for this reaction.

To ensure that MnO_2_ absorption measurements accurately reflect GOx kinetics, stability tests are also conducted by combining various other relevant chemical species and MnO_2_ nanosheets. Fig. 3a(3) shows that adding 1 mL of 1 M NaCl to a solution of MnO_2_ and DI-water causes MnO_2_ to precipitate (see inset), while Fig. 3a(2) shows that adding 1 mL of 1 M hydrogen chloride, HCl (36.5–38%, ACS Grade, ACP Chemicals H-6100) does not. The stability of MnO_2_ in DI-water (pH 7) and in HCl (pH 0) indicates that the precipitation in Fig. 3a(3) can be attributed to Na^+^ ions. Previous studies^23^ have reported that nanosheets can possess negative charges caused by Mn vacancies. We speculate that metal cations cause precipitation by interacting with such negative charges, in turn generating inter-nanosheet attraction. Precipitation is observed also when high concentrations (over 150 mM) of sodium phosphate buffer of pH 6.60–6.80 (Sigma Aldrich P8165) are added to MnO_2_ and DI-water, likely for the same reason. Accordingly, only low concentrations of phosphate buffer are used for kinetic measurements in the present study. MES buffer (a Good's buffer) yields more stable MnO_2_ solutions even at high buffer concentrations and is used for kinetic measurements over a wider range of buffer concentrations. Solutions with MnO_2_ and MES buffer in DI-water continue to remain stable upon addition of glucose (Fig. 3a(4)) or gluconic acid (49–53% by weight in water, Sigma Aldrich G1951 1 kg bottle) (Fig. 3a(5)). The range of pH over which MnO_2_ is stable includes values at which blood tests are conducted, around 5.8–7.4.^24^

**Fig. 4:**
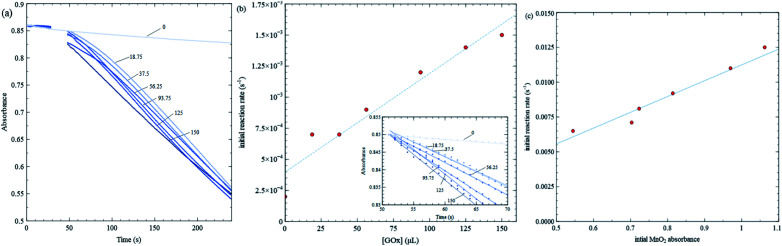
Dependence of initial rate on volume of GOx injected into a 4 mL solution in a UV-Vis cuvette, keeping nanosheet, glucose and MES buffer concentrations fixed (nanosheet concentration using the absorbance, [glucose] = 0.5 mM, [MES] = 100 mM, [GOx] = 7.5 mg mL^−1^ concentrate before injection). (a) Absorbance at 384 nm *vs.* time for various volumes of GOx injected. GOx is injected at the 30 s mark and stirred. The label for each data set indicates volume (μL) of GOx solution injected. (b) Initial reaction rate *vs.* volume of GOx injected; the plot in (b) is obtained using data shown in (a), data translated to start at the same absorbance for clarity. (c) Initial reaction rate *vs.* MnO_2_ absorbance and a linear fit.

**Fig. 5:**
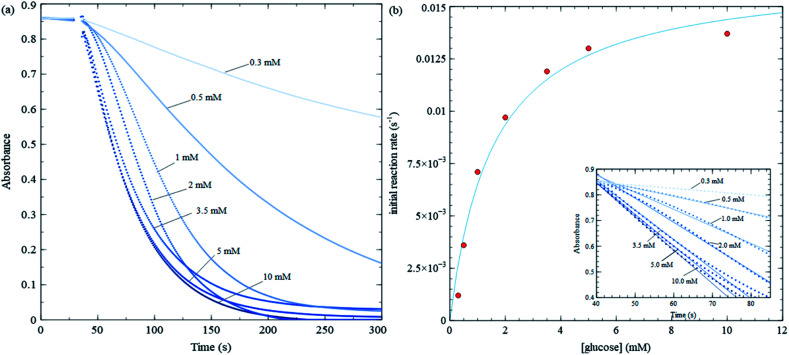
Michaelis–Menten trial with varying glucose concentration, while keeping MES buffer, GOx and nanosheet concentrations fixed. (a) Absorbance at 384 nm *vs.* time for various glucose concentrations. (b) Initial reaction rate *vs.* glucose concentration for data shown in (a), along with a least square fit to eqn (1). The fitting yield 75 μL of 7.5 mg mL^−1^ GOx is injected into a 4 mL solution in a UV-Vis cuvette at the 30 s mark and stirred.

Fig. 4 shows kinetic data varying GOx volume and initial MnO_2_ concentration. Fig. 4a shows 384 nm absorbance of MnO_2_ nanosheets *vs.* time for various GOx volumes keeping initial MnO_2_ absorbance fixed (close to 0.9). In all trials, the total solution volume is 4 mL. GOx is injected at about the 30 s mark, and the solution is stirred for 10–15 seconds. Final GOx concentrations range from 0 to 150 mM after mixing. There is a high density of data points initially, so in order to discern slopes more easily, the inset in Fig. 4b plots initial kinetic data, after fitting a straight line and removes respective offsets. Fig. 5b is analyzed similarly. The slopes (*i.e.* initial rates) are plot in the main panel. Fig. 4b shows that the initial rates *vs.* GOx volume exhibits a linear relationship (linear correlation coefficient, *R*^2^ = 0.939) consistent with the Michaelis–Menten model, as discussed further below. Subsequent kinetics measurements employed a GOx concentration that is midway in the range shown. It should be noted that there is a steady background decline in absorbance even when no GOx is added. However, this background decay occurs at a constant rate which is less than 2 × 10^−4^ s^−1^, *i.e.* 10–15 times smaller than the rates obtained for the Michaelis–Menten experiments where GOx concentration is fixed and glucose concentration is varied (see Fig. 5); hence, this background decay should not have a significant effect on the results concerning enzyme kinetics.

Fig. 4c shows initial rates for various MnO_2_ concentrations keeping all other quantities fixed. The data exhibit a linear trend, indicating the rate is first order in MnO_2_ concentration.

Fig. 5 shows kinetic data for several glucose concentrations from 0.1 mM to 10 mM, keeping MES buffer, GOx and MnO_2_ concentrations fixed. These data show that the reaction in Scheme 2 proceeds more rapidly than that in Scheme 3, and hence, changes in nanosheet absorbance can be effectively used to measure H_2_O_2_ production and GOx reaction rates. The initial reaction rate for the control test in Fig. 3b is about 0.022 s^−1^, while the fastest reaction rate over all MM trials is approximately 0.015 s^−1^. The former is sufficiently fast that a significant portion of the reaction occurs during the initial stirring period. Also, in the control test, nanosheets react with the maximum concentration of H_2_O_2_ that is injected at one time, while during MM trials, the nanosheets react with H_2_O_2_ that is produced continuously and much more slowly.

**Scheme 4:**
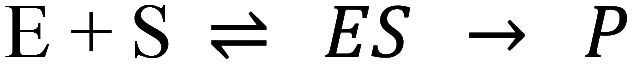


Initial rates in Fig. 5 increase (slopes become steeper) as glucose concentrations increase; however, the increases are much more pronounced at lower glucose concentrations compared with higher concentrations. The MM model predicts that the initial rate (*v*) *vs.* substrate concentration ([S]) follows the following relation:1
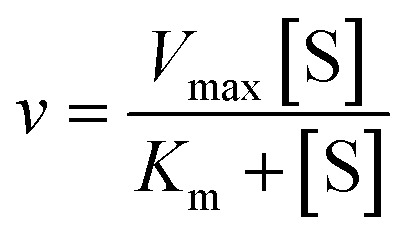
where *V*_max_ and *K*_m_ are independent of [S]. *V*_max_ = *k*_cat_[E]_0_ is the maximum initial rate achieved at high substrate concentration, *k*_cat_ is the catalytic rate constant to produce product (P in Scheme 4), and [E]_0_ is the enzyme concentration. Consistent with Fig. 4b, *V*_max_ is proportional to enzyme concentration according to the model, as mentioned. *K*_m_ is the substrate concentration at which 
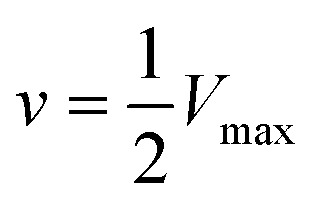
 and is a measure of enzyme performance: higher *K*_m_ values indicate lower performance since higher substrate concentrations are required to achieve 
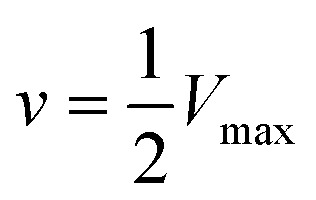
. Eqn (1) predicts that the initial rate increases linearly at low [S], when enzyme is available, and saturates to *V*_max_ at high [S], when total enzyme activity becomes a bottleneck for the reaction. Fig. 5b shows initial rates *vs.* glucose concentration, where the initial rates are calculated by linear fits to data in Fig. 5a over a 30–60 second duration following enzyme addition. Also shown is a fit to these data using eqn (1). Trends in the data are in good agreement with the MM model, both at low and high glucose concentrations.

**Fig. 6:**
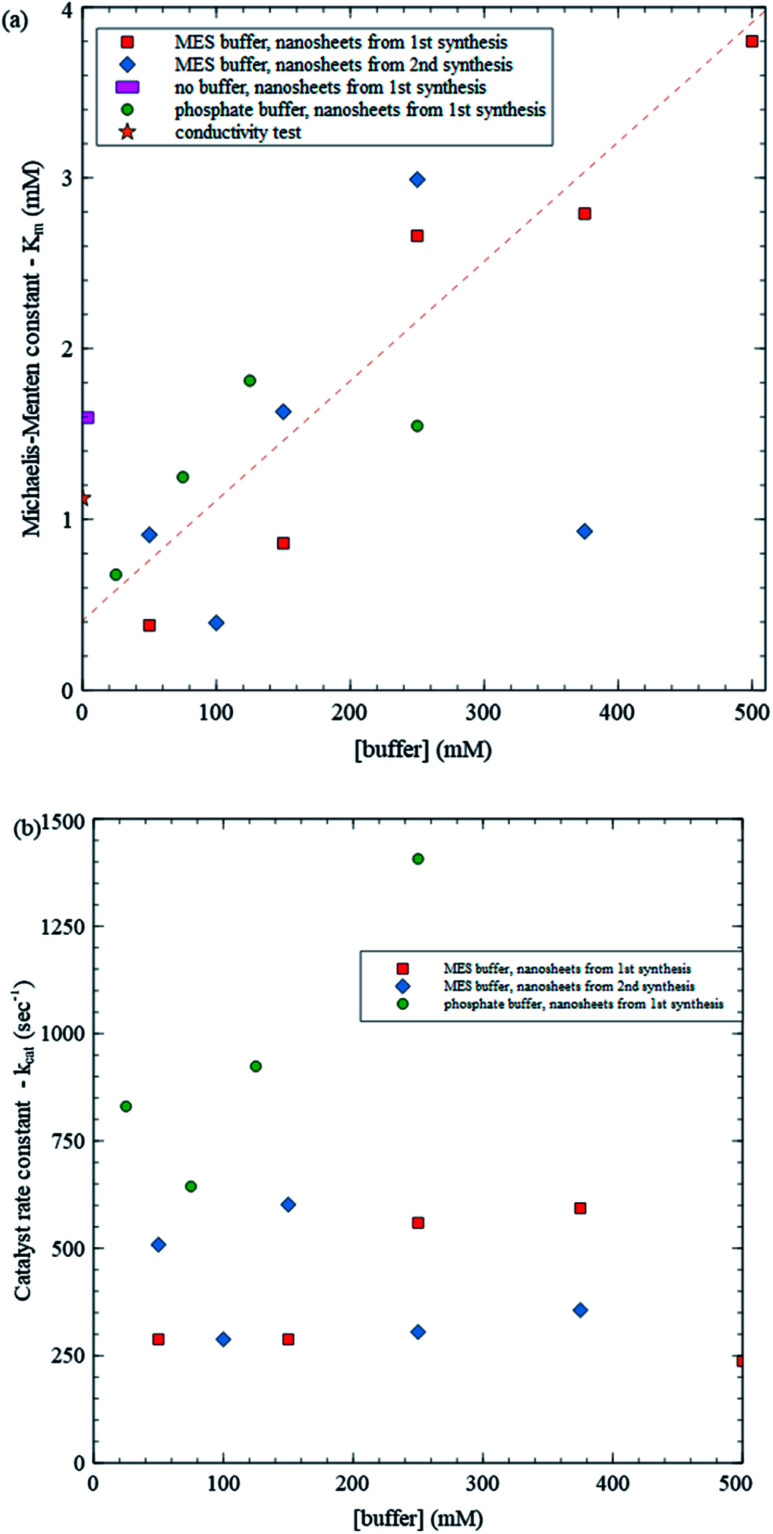
(a) Michaelis–Menten constant (*K*_m_) *vs.* buffer concentration, (b) catalyst rate constant (*k*_cat_) *vs.* buffer concentration.

As an application of the present method, we study enzyme kinetics at different buffer concentrations (all with pH ∼ 5.8) to explore any potential influence of buffer concentration on enzyme–substrate interactions. Fig. 6 summarizes the results obtained over 16 mM trials using MES buffer and nanosheets from two syntheses, phosphate buffer at low buffer concentrations and no buffer. Although the no buffer values provide a consistency check, they are expected to be less reliable due to changing enzyme activity caused by changing pH. As an independent consistency test, we also study kinetics at zero buffer concentration using a conductivity meter and include the results in Fig. 6. Production of gluconic acid (*i.e.* H^+^ ions, which are very mobile) produces a change in conductivity that is easily detected using a conductivity meter when background ion concentration is low (see Scheme 2 and Fig. S1 in ESI†). Fig. 6a shows a plot of *K*_m_*vs.* buffer concentration. A straight line fit to the data, excluding a point with *K*_m_ ≈ 1 mM at 375 mM buffer concentration and the points with no buffer, yields a reasonably good fit with *R*^2^ = 0.801 and *σ*_rms_ = 0.467, where *σ*_rms_ is the root mean square deviation from the fit. *σ*_rms_ provides a reasonable measure of variations within the data. Variations in *K*_m_ using (1) MES buffer and nanosheets from a given synthesis, (2) MES buffer and nanosheets from different syntheses and (3) different types of buffers (MES *vs.* phosphate) are all comparable to *σ*_rms_, while the excluded data point is 4.5 *σ*_rms_ from the fit suggesting that it is an outlier. As the buffer concentration increases from 20 mM to 500 mM, the trendline indicates that *K*_m_ increases from 0.5 mM to 3.9 mM, *i.e.* by about an order of magnitude. The large increase is over 7 times *σ*_rms_ indicating that it statistically significant. There is some variation between the results from the two syntheses, but it is similar to the variation within each synthesis. After removing the outlier, the *σ*_rms_ for MES synthesis 1, MES synthesis 2 and phosphate buffer are 0.35, 0.41 and 0.308 respectively, all comparable to the overall *σ*_rms_ of 0.467.

Using the *V*_max_ values from the same trials, we can determine *k*_cat_ values as well (Fig. 6b). In contrast with *K*_m_, *k*_cat_ does not exhibit a clear variation with buffer concentration: all points except one are within about one *σ*_rms_ (313 s^−1^) from the average (559 s^−1^). Combined, these results provide some insight into how varying buffer concentration affects enzyme kinetics. According to the MM model,
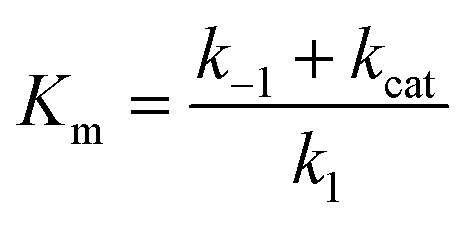
where *k*_1_ and *k*_−1_, respectively, are the rate constants for the forward and reverse reactions of the enzyme + substrate ⇋ enzyme–substrate complex reaction (Scheme 4). In order for *K*_m_ to increase while *k*_cat_ remains constant with increasing buffer concentration, the enzyme–substrate complex should decompose back to reactants more quickly (*k*_−1_ increases) and/or the enzyme–substrate complex should form more slowly (*k*_1_ decreases).

As mentioned, trends in *K*_m_ and *k*_cat_ with buffer concentration have not been reported in the literature. Reported values of *K*_m_ range widely across various studies from just under 10 to over 100 mM, and *k*_cat_ values vary between 100 to 1000 s^−1^.^25–31^ Reported *K*_m_ values are about an order of magnitude larger than those found in the present study and vary over an order of magnitude. Fig. 6a shows that differences in buffer can lead to large variations in values of *K*_m_, suggesting that this may contribute to variations in reported values of *K*_m_ and that significantly lower *K*_m_ (and correspondingly higher enzyme performance) can be achieved depending on the buffer. Interestingly, *k*_cat_, values found here are similar in magnitude to reported values, which is consistent with *k*_cat_ not exhibiting a systematic variation with buffer concentration.

The use of MnO_2_ nanosheets enables a straightforward analysis of GOx kinetics and yields good agreement between the observed kinetics and the MM model. It also enables exploring the influence of varying buffer concentration. Previous studies have reported that buffer concentration has an effect on protein and enzyme structures, causing them to denature.^32^ Given such denaturing (and potentially also interference with binding to other species, or mechanisms for degrading operation), loss of function can be anticipated in environments with high ion concentration. The present results suggest that similar adverse effects can be anticipated for buffer–enzyme combinations as well. To our knowledge, this is the first study to quantify a large, linear increase in *K*_m_, indicating degrading function, with increasing buffer concentration using GOx as a test bed. These results are significant because they suggest that, whereas having some buffer is desirable to keep pH and enzyme activity stable, a minimum of buffer should be used to prevent significant adverse impact on enzyme performance.

## 4. Conclusions

Decomposition of MnO_2_ nanosheets by H_2_O_2_ and UV-Vis real time spectroscopy are used to study GOx kinetics. The study finds that GOx kinetics follows the MM model. Also *K*_m_ increases approximately linearly by about an order of magnitude with increasing buffer concentration. These results demonstrate the facility of the present method for studying GOx kinetics, and the strong adverse effect of high buffer concentrations on GOx and potentially other enzyme-catalyzed reactions.

## Conflicts of interest

There are no conflicts to declare.

## Supplementary Material

NA-003-D1NA00311A-s001
