# Supplementary material for: Glucose oxidase kinetics using MnO2 nanosheets: confirming Michaelis–Menten kinetics and quantifying decreasing enzyme performance with increasing buffer concentration
Source: Nanoscale Adv. 2021 May 17;3(13):3816–23. doi: 10.1039/d1na00311a (PMC9419709; doi:10.1039/d1na00311a)
Supplement: NA-003-D1NA00311A-s001 [file NA-003-D1NA00311A-s001.pdf]

Electronic Supplementary Material (ESI) for Nanoscale Advances.
This journal is © The Royal Society of Chemistry 2021

Nanoscale

Electronic Supplementary Information (ESI)

Received 00th January 20xx,
Accepted 00th January 20xx

DOI: 10.1039/x0xx00000x

Glucose Oxidase Kinetics using MnO_2 Nanosheets: Confirming Michaelis – Menten Kinetics and Quantifying Decreasing Enzyme Performance with Increasing Buffer Concentration

Mahip Singh, ‡ [a] Ungku Zoe Anysa Ungku Faiz, ‡ [a] Steven Gravelsins,[a] Yoshinori Suganuma,[a] Nicholas Konstantine Kotoulas,[a] Mark Croxall,[a] Ahlia Khan - Trottier,[b] Cynthia Goh,[a] and Al - Amin Dhirani,[a, c, †]

a. Department of Chemistry, University of Toronto, Ontario, Canada, M5S 3H6

b. Department of Biochemistry, University of Toronto, Ontario, Canada, M5S 3H6

c. Department of Physics, University of Toronto, Toronto, Ontario, Canada, M5S 3H6

‡ These authors contributed equally

† E-mail: a.dhirani@utoronto.ca

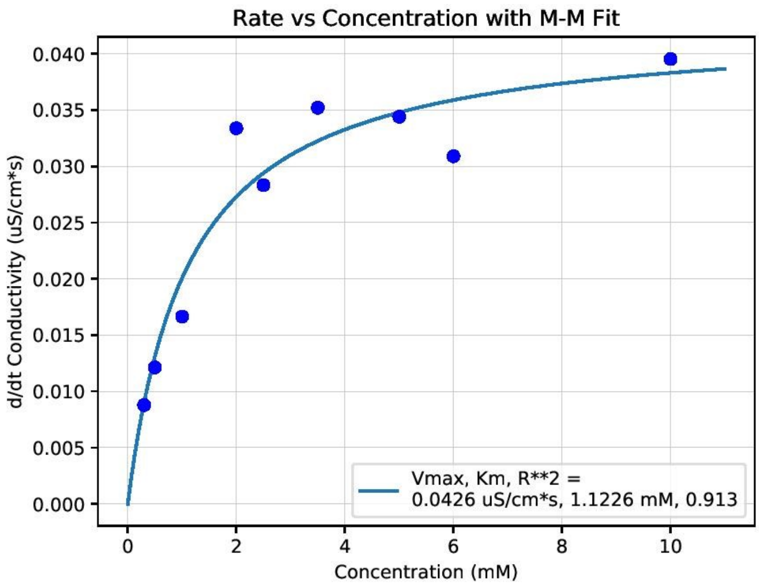

Fig. S1 Initial rate vs glucose concentration obtained by analyzing real-time kinetics data for the reaction shown in Scheme 3. The data are obtained using a UNS-Tech conductivity meter to monitor changes in conductivity arising from the production of gluconic acid (H^+) with no buffer.

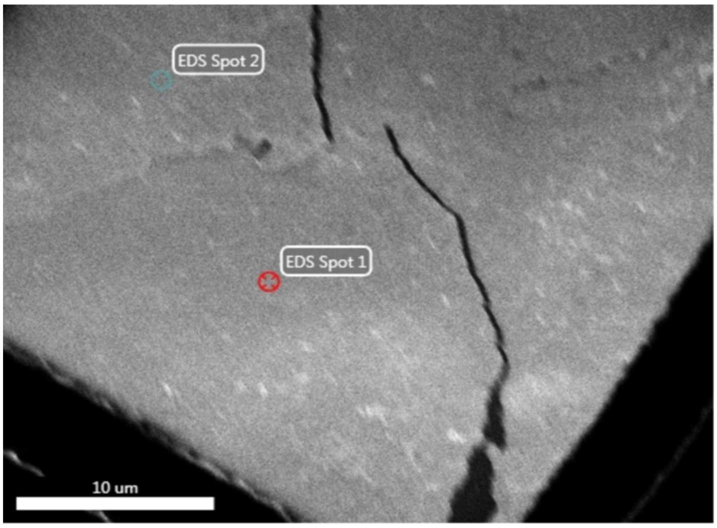

Fig. S2 ESEM image of MnO_2 Thick film used for EDS.

EDS Spot 1:

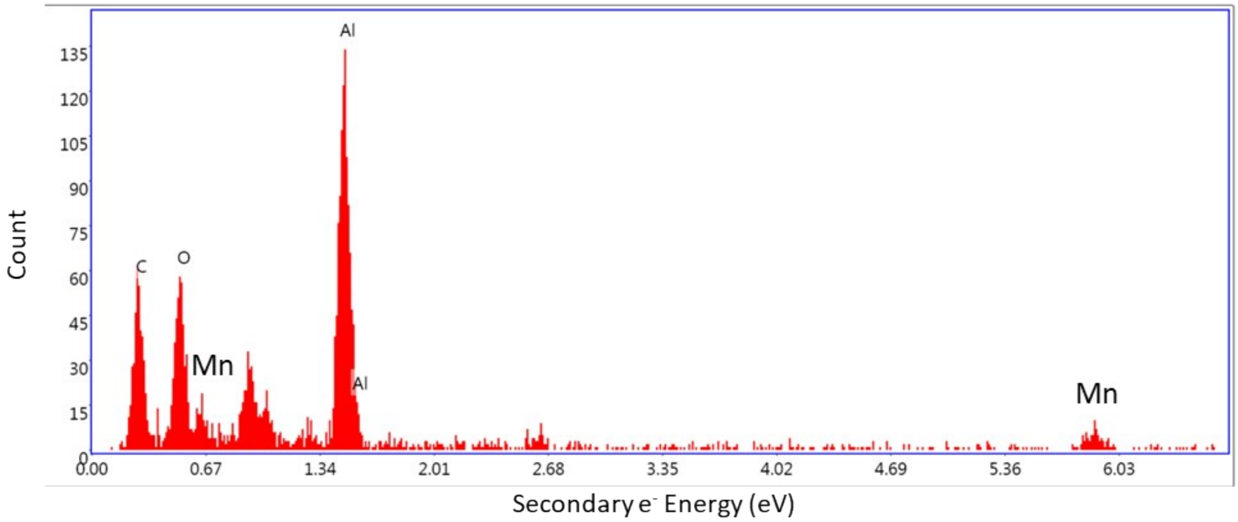

Fig. S3 EDS Spectra of MnO_2 thick film. Accelerating voltage of 10KeV was used. Al present due to sample holder, C present due to STEM grid. MnO_2 is relatively transparent to E-beam, so a thick film was needed to obtain spectra with appropriately high count. Characteristic Mn Kα seen centred at 5.9eV and Lα at 0.637eV

This journal is © The Royal Society of Chemistry 2020

Please do not adjust margins

EDS Spot 2:

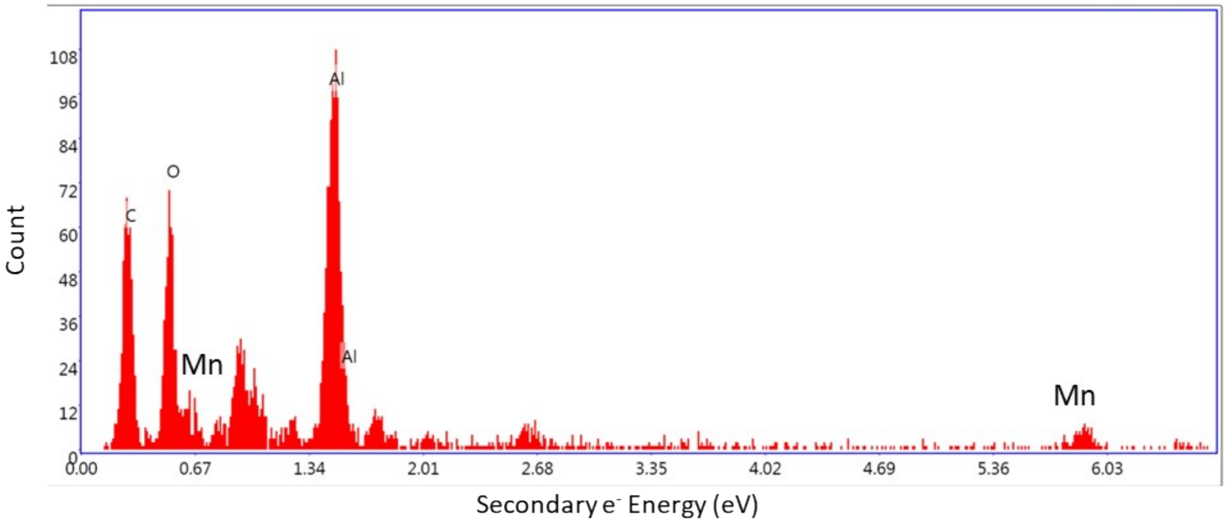

Please do not adjust margins
